# Integration of family planning services with HIV treatment for women of reproductive age attending ART clinic in Oromia regional state, Ethiopia

**DOI:** 10.1186/s12978-021-01157-0

**Published:** 2021-05-22

**Authors:** Dereje Bayissa Demissie, Rose Mmusi-Phetoe

**Affiliations:** 1 Department of Health Studies, College of Human Science, University of South Africa, Pretoria, South Africa; 2 St. Paul’s Hospital Millennium Medical College, Addis Ababa, Ethiopia; 3 Department of Public Health, College of Human Science, University of South Africa, Pretoria, South Africa

**Keywords:** Family planning, HIV services, Integration, Women living with HIV

## Abstract

**Background:**

In settings where HIV prevalence is high, management of sexual and reproductive health is critical to reducing HIV transmission and maternal mortality. Integration of family planning with HIV services is appropriate for HIV therapy, HIV prevention, and care in a resource-limited country s like Ethiopia. The study aimed at examining the status of integration of family planning services with HIV treatment and factors associated with successful integration of family planning and HIV services for women of reproductive age in Oromia, Ethiopia for better health outcomes.

**Methods:**

The research design of this study was a quantitative survey, non-experimental, explorative and descriptive. A questionnaire was used to collect data from women living with HIV attending ART clinics in the special zone of surrounding Finfinne, Oromia Region in five health centers. Simple random sampling was used to select 654 respondents. Data was analysed through the use of Statistical Package for Social Sciences version 23.0. Bivariate and multivariate logistic regressions were performed to identify factors associated integration of family planning with HIV services with the significant association at an adjusted odds ratio (AOR) with a 95% confidence interval (CI) to controlled effects of possible confounders from the final model.

**Result:**

The response rate of this study was 97.6% (654/670). The ages of those who responded to the administered questionnaires ranged between 18 and 49 years. The mean age of the respondents was 31.86 years with an SD of ± 6.0 years. Most of the respondents in the sample were in the age group 26–35 (n = 374, 57%), and only 96 (14.7%) were in the age group 18–25. This overall integration of FP-HIV services among reproductive-age women living with HIV in Oromia regional state of special zone health centers was found to be 55.8%. Almost all respondents (n = 635, 97.1%) preferred integrated family planning and HIV services from the same facility and the same providers. the study found that 622 (95%) were most satisfied with the utilization of integrated family planning/HIV services.

**Conclusion:**

This study established that in overall, the integration of family planning/HIV services was relatively moderate among women of reproductive age living HIV. The identified factors that affected the integration of family planning with HIV services were the level of education, occupational status, residence, discussion of family planning with healthcare providers, fertility desire and CD4 counts.

**Supplementary Information:**

The online version contains supplementary material available at 10.1186/s12978-021-01157-0.

## Plain language summary

These study findings are strongly in favour of integrated services offering multiple health services at the same facility by the same provider. Service integration based on the needs of women of reproductive age was found to be necessary in public health facilities where people have access only to primary healthcare facilities.

Integrated family planning with HIV (FP-HIV) services contribute to women’s wellbeing by addressing a wide range of health needs and offering convenience of receiving multiple services a single visit. Integrated FP-HIV services further address women’s vulnerability to other sexual reproductive health problems.

Integrated FP-HIV services should contribute to comprehensive national family planning programs to enable the provision of full access to a variety of contraceptive methods. The latter should enable couples and individuals to obtain services at a one-stop supermarket to achieve optimal reproductive and sexual health to meet their needs.

The findings from study generated adequate knowledge and equipped managers with skills integrating family planning with HIV services among women of reproductive age in Oromia regional state, Ethiopia.

The study’s findings have emphasized the urgent need to fully implement action focusing on integrating family planning/HIV services for women of reproductive age living HIV and attending ART clinics. The study prioritized the need to strengthen the capacity of health centers to provide quality FP-HIV.

**Supplementary Information:**

The online version contains supplementary material available at 10.1186/s12978-021-01157-0.

## Introduction

The integration of ‘family planning with human immune-deficiency virus (HIV) services to promote contraceptive use among "women living with HIV" has emerged as a rich ground for research. Integrating family planning and HIV services is a process that occurs at different levels of the health care system such as national, regional, district, and health facility levels for effective functioning of key functions such as governance, financing, planning, service delivery, monitoring, evaluation, and demand generation [1].

In this study, integration family planning with HIV treatment means linking these programs by offering one-stop comprehensive at a healthcare facility [2].

According to WHO [3] member countries should ensure universal access to sexual and reproductive healthcare services, including family planning, information and education, and the integration of reproductive health into national strategies and programs by the end of 2030 [3, 4]. According to a study by [5], integration of health service delivery is key to addressing improvements in MNCH services and HIV care and treatment in sub-Saharan African countries. Public health programs emphasize that the integration of family planning services with HIV treatment to increases dual contraceptive methods' utilization will ensure protection from both unintended pregnancy and STIs, including HIV/AIDS [6].

### Statement of the problems

In 2012 it was estimated that in sub-Saharan Africa, 53 million women who wanted to avoid pregnancy were not using any family planning method [7]. Sarnquist et al. [8] add that an unmet need for contraception among women living with HIV in sub-Saharan Africa is high, with 66–92% of women reporting not wanting another child (now or ever). Only 20–43% of these women were using contraception*.* The prevalence of unmet needs for family planning thus remains unacceptably high among women in sub-Saharan Africa, including those living with HIV, even if they are involved in HIV treatment programs [7, 8].

According to the ‘Ethiopia Demographic and Health Survey’ (EDHS) of 2016, 28.9% of currently married women in the Oromia Region have an unmet need for family planning services; 17.1% for spacing, 11.8% for limiting, and only 28.6% women have received family planning services. The total demand for family planning in Oromia Region was 57.55% [9].

Meeting the unmet needs for family planning in sub-Saharan Africa could make an important contribution to improving maternal health through early studies 2008, the estimated maternal mortality ratio in sub-Saharan Africa was 596 per 100,000 live births, the contraceptive prevalence was 22%, and the proportion of maternal deaths averted by contraceptive use was estimated at 32%. In contrast, among low-and-middle-income countries as a group, the maternal mortality ratio was 273, the contraceptive prevalence was 63%, and 44% of maternal deaths were estimated to be averted by family planning use [10].

Programs that have succeeded in promoting condom use and providing HIV prevention and treatment services in this region have largely missed the opportunity to address the contraceptive needs of the key populations they serve. Therefore, the research statement for this study what is the status of the integration between family planning and HIV treatment services for women of reproductive age living with HIV attending healthcare facilities in the Oromia Region, Ethiopia?

## Methods and materials

### Research setting and design

This study was conducted in the Oromia Region surroundings of Finfinne Oromia, Ethiopia. Currently, the health system of the zone consists of two hospitals under construction, and 27 health centers with 98% potential health service coverage. There were different governmental and non-governmental organizations working on HIV/AIDS in the zone. 13 health centers have been providing ART and family planning services in the zone, of which five were randomly selected as the study setting. The total number of people living with HIV enrolled at ART clinics in the zone was 9421, of which 2380 were women of reproductive age, and of these, 1557 were from five randomly selected health centers [11]. The target population was HIV-positive women of reproductive age who had attended ART follow-up services for at least six months from randomly selected healthcare facilities in the Oromia Region, Ethiopia. The accessible sample was 1557 eligible women of reproductive age living with HIV attending ART clinics in public health centers.

A quantitative survey, non-experimental, explorative, and descriptive. A questionnaire was used to collect data from women living with HIV attending ART clinics.

### Sample size determination

The sample size was determined by using single population proportion formula with the assumption that 40% proportion (P) of women of reproductive age living HIV have utilized family planning at integrated sites of Health facility in Ethiopia [12] at a 5% level of significance and a margin of error of 5%. By considering the design effect of 2, with correction formula since the total population was less than 10,000 (2380) and with a 5% non-response rate considered, the final sample size was 670 women living with HIV.

### Sampling procedure

All hospitals and health centers found in the Special Zone of Oromia Region that provide ART services were identified and randomly selected by computer-generated methods by using SPSS version 23.0 software application to be included in the study. Study sites were prepared and entered into SPSS version 23 by using their pre-ART registration numbers from the health management information system (HMIS) database. A list of all women living with HIV from each facility, aged between 18 and 49 years of age, was randomly created. Simple random computer-generated sampling was utilized at each health center to select 670 study respondents. The number of study respondents was allocated proportionally for the five health centers, based on their total number of ART clients.

### Data collection

The questionnaire used for data collection was initially prepared in English, and translated to Afan Oromo, and back to English for language experts to confirm its consistency. Finally, the corrected Afan Oromo version was used to collect the data from women living with HIV attending ART clinics. The questions included in the questionnaire were adapted and prepared by reviewing different related literature and variables identified to be measured. The training was given to data collectors and supervisors by the primary researcher for two days. Data collectors cross-checked the pre-ART card numbers of women living with HIV who came to the ART clinic with sampled card numbers daily. Five trained data collectors collected data from women of reproductive age. The completed questionnaires were collected and checked daily for consistency and completeness by supervisors and the primary researcher. Data were collected using a pre-tested structured Afan Oromo version of the questionnaire. A pre-test of the questionnaire was done on 5% of the women living with HIV at Ambo health center, to identify any ambiguity, to confirm consistency in the questionnaire, to determine acceptability, and to make necessary corrections one week before the actual data collection process. The respondents were guided through a questionnaire and chart abstraction conducted at their health facility by trained data collectors.

### Operational definition

Integration of family planning with HIV treatment is offered at the same facility during the same operating hours, and the provider of one service encourages clients to consider using the other service during that visit which implies the possibility of more than one location within the facility and more than one service provider. In this study, integration of family planning with HIV treatment means that organizing these program by offering one-stop comprehensive and integrated services at ART Room [2]. The study objective of this to examine the status of integration of family planning services with HIV treatment for women of reproductive age in Oromia, Ethiopia.

The ten measurement variables related to integrating family planning/HIV services were analyzed through SPSS under data transform count the occurrence of a value in terms of the respondents who answered "yes" to the integration of family planning/HIV services.

### Data management and analysis

The returned questionnaires were checked for completeness, cleaned manually, coded and entered into EPI INFO 7.1.6 version, and then transferred to SPSS version 23 for further analysis. Frequencies, percentages, mean and standard deviation (SD) were used to summarise descriptive statistics of the data and text. Moreover, tables and graphs will be used for data presentation. Bivariate analysis was used primarily to check which variables have an individual association with the dependent variable. In this analysis, the outcome variables, integrated family planning/HIV services, were dichotomized with "1" being integrated and "0" not integrated. Two different models were employed to investigate the factors predicting integration of family planning/HIV services. Accordingly, the Hosmer–Lemeshow Test (HL) for the two models showed chi-square p-values > 0.05, which proved the goodness-of-fit of the applied models for this study at p = 0.56 for the integrated family planning/HIV services model.

The estimates of the crude and adjusted odds ratio (AOR) were fairly similar and this showed that the variables used for adjustment were not confounding variables [13]. Variables that were found to have an association with the dependent variables were then entered into multiple logistic regressions to control the possible effect of confounders. Finally, the variables which have significant association were identified based on AOR, with a 95% CI and p-value to fit into the final regression model.

## Results

### Response rate

The response rate is the number of participants who completed a questionnaire, after 16 spoiled questionnaires were discarded. The study achieved a response rate of 97.6% 654/670. The latter reflects the quality of training provided to interviewers, their understanding, and the daily supervision by the principal investigator.

There were 654 respondents whose ages ranged between 18 and 49 years. The mean age of the respondents was 31.86 years with an SD of ± 6.0 years. Most of the respondents in the sample were in the age group 26–35 (n = 374, 57%) as reflected in Table 1.

**Table 1:** Demographic and socioeconomic characteristics of respondents in Oromia Region, Ethiopia 2018 (N = 654)

Demographic and social characteristics	Category	Frequency (%)	Cumulative (%)
Age in year (n = 654) Mean (SD): 31.86 (± 6.0)	18–25	96 (14.7)	14.7
	26–35	374 (57.2)	71.9
	36–49	184 (28.1)	100.0
Ethnic group	Oromo	460 (70.3)	70.3
	Amhara	155 (23.7)	94.0
	Tigre	10 (1.5)	95.6
	Gurage	26 (4.0)	99.5
	Others (Wolayita, sidama)	3 (0.5)	100.0
Highest level of education	Never been school	245 (37.5)	37.5
	Primary	284 (43.4)	80.9
	Secondary	106 (16.2)	97.1
	College/university	19 (2.9)	100.0
Marital status	Married	528 (80.7)	80.7
	Cohabit/living together	51 (7.8)	88.5
	Divorced/separated	46 (7.0)	95.6
	Widowed	22 (3.4)	98.9
	Single	7 (1.1)	100.0
Religious affiliation	Orthodox	474 (72.5)	72.5
	Protestant	131 (20.0)	92.5
	Muslim	42 (6.4)	98.9
	Catholic	7 (1.1)	100.0
Family monthly income (1$ = 27.84 Birr)	< = 800 Ethiopia Birr	166 (25.4)	25.4
	801–1200 Ethiopia Birr	191 (29.2)	54.6
	1201–1800 Ethiopia Birr	136 (20.8)	75.4
	1801 + Ethiopia Birr	161 (24.6)	100.0
Employment status	Government employee	59 (9.0)	9.0
	Merchant/private work	239 (36.5)	45.6
	Housewife	256 (39.1)	84.7
	Farmers	55 (8.4)	93.1
	Unemployed	45 (6.9)	100.0
Residence	Urban	518 (79.2)	79.2
	Rural	136 (20.8)	100.0

Table 1 presented the education status of the respondents which revealed that the literacy proportion was 409 (62.5%). The majority (n = 409, 62.5%) of the respondents had at least attended school from primary level to college/university level, and the least represented were 19(2.9%) who had attained tertiary level education in the form of attending a college or university. In terms of religious affiliation, 474(72.5%) respondents belonged to the orthodox denomination,

Of the 609 (93.1%) employed respondents, 256 (39.1%) were housewives. These were followed by 239 (36.5%) who were in the private or merchant sector, 55 (8.4%) were self-employed in agriculture on their farms, and only 59 (9.0%) were working for the public service sector. The family’s monthly income distribution among the respondents was assessed, and it was found that on average, the income was 1398.18 Ethiopian Birr (50$), and ranged from 100 to 5000. More than 357 (54.6%) respondents were earning less than 1201 Ethiopian birr (1$ = 27.84Birr).

### Integrating family planning with HIV services

On assessing the level of integrated family planning with HIV services in the ART clinics, this study found that the ART providers provided a contraceptive method mix in ART clinics, of which 93.7% were condoms, 90.2% were injectable and 82.3% were oral contraceptives as chosen methods available during the study period. Therefore, the family planning/HIV services were integrated with the ART clinics of the Oromia Region and specifically focused on offering counseling on available family planning services to providing injectable contraceptive methods, pills, and condoms in the ART clinics. The integrated family planning/HIV services also referred women of reproductive age for consultation on available long-acting reversible and permanent methods within the same facility.

Of the total number of respondents, 355 (54.3%) received family planning counseling on available contraceptive methods by trained health professionals in the waiting room. Moreover, 548 (83.8%) had attended family planning health education sessions in service settings offering ART, PMTCT, STI, VCT, and tuberculosis services. Of the total number of women of reproductive age living with HIV, 506 (77.4%) had received family planning counseling on the efficacy of each method, its side effects, and method mix available in addition to ART services. Based on the counseling mentioned, a notable number of women living with HIV were referred for consultation at the family planning unit within the same facility on available long-acting and permanent contraceptive methods. The study revealed that 450 (68.8%) were referred for implants, 401 (61.3%) for an IUD, and 190 (29.1%) for tubal ligation. The study further revealed that 548 (83.8%) women living with HIV had received dual protection information during counseling, of which 337 (51.5%) accepted dual method contraceptives from ART providers to prevent both unintended pregnancy and HIV transmission.

Table 3 depicts that 422 (64.5%) women living with HIV who were attending ART were screened, counseled, and provided with injectable contraceptives, and 151 (23.1%) received an implant during their ART drug refilling at the clinic.

Of the respondents, 616 (94.2%) mentioned that service providers were knowledgeable and comfortable in providing integrated family planning/HIV counseling, and 537 (82.1%) stated that service providers were knowledgeable and comfortable providing integrated family planning/HIV services. Table 2.

**Table 2:** Integration of family planning/HIV services of health Centres in Oromia Region, Ethiopia 2018

Level of integration of family planning/HIV services	Categories	Frequency (%)		
Receipt of family planning counselling in the waiting room	Yes	355 (54.3)		
	No	299 (45.7)		
Choice of contraceptive methods in need of available methods		*Yes*		*No*
	Injectable	590 (90.2)		64 (9.8)
	Condoms	613 (93.7)		41 (6.3)
	Oral contraceptives	538 (82.3)		116 (17.7)
Referral of clients for consultation of on available long-acting and permanent methods	Implants	450 (68.8)		204 (31.2)
	Intrauterine device (IUD)	401 (61.3)		253 (38.7)
	Tubal ligation	190 (29.1)		464 (70.9)
	Vasectomy	167 (25.5)		487 (74.5)
Counselling about each method efficacy, side effects and available contraception mix in addition to ART services		Yes	506 (77.4)	
		No	148 (22.6)	
Attended family planning health education sessions in service settings offering ART, PMTCT, STI, VCT and tuberculosis services		Yes	249 (38.1)	
		No	405 (61.9)	
Information provided on dual protection during ART drug refill in the ART room		Yes	548 (83.8)	
		No	106 (16.2)	
Dual method contraceptive provided for prevention of both unintended pregnancy and HIV transmission		Yes	337 (51.5)	
		No	211 (32.3)	
Screening, counselling and provided injectable family planning in the ART room		Yes	422 (64.5)	
		No	232 (35.5)	
Screening, counselling and provided implant for clients in the ART room		Yes	151 (23.1)	
		No	503 (76.9)	
provided instructions of IUD or implant, including recommended date of removal provided		Yes	412 (63.0)	
		No	242 (37.0)	
Counselling offered for informed decision-making and consent for permanent methods		Yes	524 (80.1)	
		No	130 (19.9)	
Service providers knowledgeable and comfortable with providing integrated family planning/HIV counselling		Yes	616 (94.2)	
		No	38 (5.8)	
Service providers knowledgeable and comfortable with providing integrated family planning/HIV services		Yes	537 (82.1)	
		No	117 (17.9)	

**Fig. 1:**
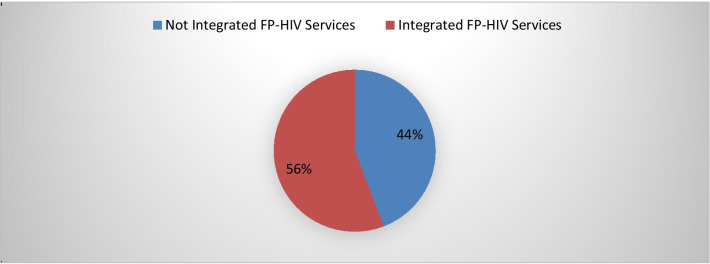
Integration family planning/HIV services for women living with HIV attending ART clinics in Oromia, Ethiopia 2018

The overall integration of family planning/HIV services was reported by 365 (55.8%) of 654 respondents, which ranges from 51.8 to 59.5% with 95%CI (Fig. 1).

**Fig. 2:**
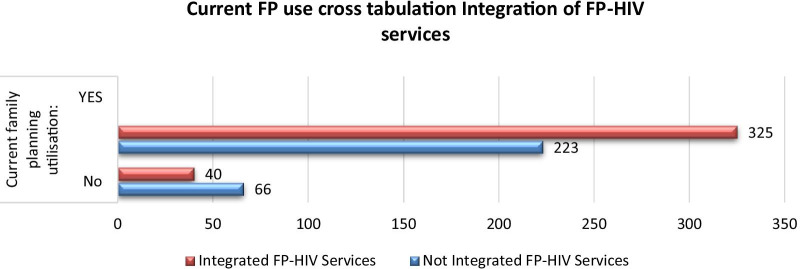
Distribution of current family planning using cross tabulation

Figure 2 reveals that as integrated family planning/HIV services increased, the number of modern contraceptive utilisers also increased. It was discovered that 325 (50%) current family planning users were using integrated family planning/HIV services versus 40 (6.1%) who were not using integrated services (Fig. 2).

**Fig. 3:**
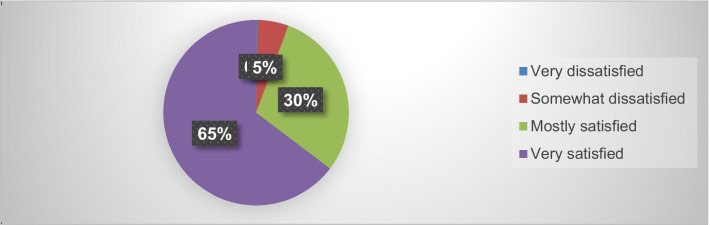
Level of satisfaction on the utilisation of integration of family planning/HIV services in Oromia, Ethiopia, 2018

This study determined that the integration of family planning with HIV services ranged from counseling on family planning in the ART room to the provision of injectable contraceptive methods. Moreover, it also entailed patients being referred to a family planning unit in the same facility for long-acting and permanent contraceptive methods. An exit interview was conducted to determine the level of satisfaction on the utilization of integrated services, as briefly exhibited in Fig. 3. The exit interview results revealed that more than 622 (95%) respondents are very or mostly satisfied with the utilization of integrated family planning/HIV services.

In this study, almost all respondents (n = 635, 97.1%) preferred integrated sexual reproductive health and HIV services at the same facility, from the same providers, and 622 (95%) were very or mostly satisfied with the utilization of integrated family planning/HIV services.

### Factors associated with the integration of family planning/HIV services

Bivariate analysis was used primarily to check which variables had an individual associated with the dependent variable.

Variables that had significant association were identified based on an AOR with 95%CI and p-value to fit into the final regression model.

Table 3 depicts variables associated with the integration of family planning/HIV services by multivariable logistic regression.

**Table 3:** Factors associated with Integration of family planning/HIV services at multivariable logistic regression (AOR, 95% CI) in Oromia, Ethiopia 2018

Factors associated with Integration of family planning/HIV services		Integration of family planning/HIV services		COR (95% CI)	AOR (95% CI)
		Yes	No		
Attended school: Yes		272 (41.6)	137 (20.9)	3.25 (2.33–4.51)***	1.73 (1.09–2.73)*
No		93 (14.2)	152 (5.7)	1:00	1:00
Occupational: Gov't		46 (7.0)	13 (2.0)	3.69 (1.58–8.65)**	5.16 (1.67–15.94)**
Merchant/private work		156 (23.9)	83 (12.7)	1.97 (1.034–3.74)*	2.79 (1.18–6.58)*
Housewife		125 (19.1)	131 (20.0)	0.998 (0.53–1.88)	1.31 (0.57–3.00)
Farmer		16 (2.4)	39 (6.0)	0.429 (0.188–0.98)*	1.23 (0.419–3.62)
Unemployed		22 (3.4)	23 (3.5)	1:00	1:00
Residence: Urban		325 (49.7)	193 (29.5)	4.04 (2.68–6.087)***	2.61 (1.47–4.62)***
Rural		40 (6.1)	96 (14.7)	1:00	1:00
Discussed with healthcare provider	Yes	344 (52.6)	199 (30.4)	7.4 (4.47–12.29)***	5.83 (3.07–11.06)***
	No	21 (3.2)	90 (13.8)	1:00	1:00
Fertility desire	Yes	212 (32.4)	112 (17.1)	1:00	1:00
	No	153 (23.4)	177 (27.1)	2.19 (1.598–3.0)***	1.804 (1.156–2.82)***
Family planning Counselled	Yes	291 (44.5)	64 (9.8)	13.8 (9.48–20.16)***	14.69 (9.36–23.1)***
	No	74 (11.3)	225 (34.4)	1.00	1:00
Recent CD4 cells/ml^3^ count	< = 350 cells/ml^3^	72 (11.0)	92 (14.1)	1:00	1:00
	351 to 500 cells/ml^3^	95 (14.1)	65 (9.9)	1.87 (1.2–2.90)**	1.57 (0.867–2.83)
	> = 501 cells/ml^3^	198 (30.3)	132 (20.2)	1.92 (1.31–2.80)***	1.82 (1.087–3.047)*

*CI* confidence interval, *AOR* adjusted, *COR* crude odd

***p < 0.001, **p < 0.01, *p < 0.05

Table 3 shows the final regression model which indicates that N = 654 (p < 0.019), attended school (AOR 1.73, 95% CI 1.09–2.73, p = 0.004), had an occupational status with the government (AOR 5.16, 95% CI 1.67–15.94 and p < 0.019), merchant/private work (AOR 2.79, 95% CI 1.18–6.58) compared to those who were unemployed (p < 0.001), in urban residence (AOR 2.61, 95% CI 1.47–4.62, p < 0.000), discussed family planning with a healthcare provider (AOR 5.83, 95% CI 3.07–11.06, p < 0.009), had fertility desire (AOR 1.804, 95% CI 1.156–2.82, p < 0.000), were counselled on family planning (AOR 14.69, 95% CI 9.36–23.07, p < 0.023) had a recent CD4 cells/ml^3^ of 501 and above (AOR 1.82, 95% CI 1.087–3.047). These factors were independently associated with increased integration of family planning/HIV services.

## Discussion

This study determined the overall integration of FP-HIV services was 55.8% among reproductive-age women living with HIV with identified factors that affected the integration of family planning with HIV services were educational and occupational status, residence, discussion of family planning with healthcare providers, fertility desire, and CD4 counts.

The socioeconomic characteristics of the respondents as summarised in Table 1 are not different from the socioeconomic profile of Ethiopia. For example, in the general population of the same region, Christian denominations dominate and represent 65% of the population, 79.2% resided in urban areas, and the largest ethnic group is Oromo, followed by Amhara which represent 64% of the population [9]. The results are also similar in terms of the proportion of women who are currently married or living together with a partner (65%) in the general population [9].

### Integration of family planning services with HIV treatment in Oromia

The study conducted by Bradley et al. [14] on VCT and family planning service integration in Ethiopia showed that counselors jointly offered HIV and family planning services with many repeat family planning clients. These health facilities attracted both standard MNCH clients [14]. On the integration of family planning services with HIV and related factors among those who came for VCT, the study established that the services offered were limited to information sharing on family planning methods and ARTs as well as referrals only.

The study by Bradley et al. [14] could not establish any integration of family planning services with HIV and related factors (family planning-HIV integration services) in Ethiopia in general, nor in Oromia in particular. This thesis gives a more comprehensive representation of the integration of family planning services with HIV services and its relationship with other selected factors in Oromia, hence determining the level of integration with sexual reproductive health, family planning, PMTC, and ART services. The integration includes a combination of training offered to service providers, supervision, and services provided by healthcare providers.

As far as the integration of family planning and HIV services is concerned, the study revealed that the ART clinics provided both ART drugs and contraceptive methods in ART clinics, of which 93.7% were condoms, 90.2% were injectable, and 82.3% were oral contraceptives as chosen methods available during the study period. These findings were supported by a systematic review by O’Reilly et al. [15], who claim that concerted efforts on the provision of information and support for family planning use, coupled with ready access to a wide range of contraceptive methods, seemed to be most effective in increasing family planning utilization.

The proportion of contraceptive information that was provided and utilization of the ART clinics was higher in this study, compared to a study conducted in Ghana. That study reported that 74% of women living with HIV had received information on contraception, 42.6% of participants and/or their partners were using a contraceptive method, and 79.6% used condoms [16]. The differences may be due to sociodemographic factors and the different healthcare systems in the two countries. For example, the training modules of family planning and HIV services were integrated in Ethiopia in line with an emphasis at the global level on SDGs. These strongly recommend the integration of sexual reproductive health services with HIV services [3]. One can conclude that the integration of family planning/HIV services in the Oromia Region, Ethiopia is more comprehensive as it ranged from counseling on available family planning services to the provision of injectable contraceptive methods, oral contraceptive pills, and condoms in the ART clinics. Integration also includes referring women of reproductive age for consultation on available long-acting and permanent methods on family planning within the same facility.

As mentioned, this study showed that 93.7% of respondents used condoms, 90.2% injectable, and 82.3% oral contraceptives. Moreover, 54.3% received family planning counseling on available methods. The contraceptive methods were provided by trained health professionals in the waiting room. A further revelation in this study is that 77.4% of respondents received family planning counseling about the efficacy of each method and the clients were referred to a family planning unit within the same facility for consultation on available long-acting and permanent methods, of which 88.8% received an implant, 61.3% IUDs and 29.1% tubal ligation.

This study’s findings are similar to those of the study conducted in Lusaka by [17] which revealed that 80% of respondents accessed family planning services in ART clinics, and 99% reported having used modern contraception, of which 60% used condoms, 15% injectable contraception, and 11% oral contraceptive pills. This result indicates that healthcare providers should offer a standardized family planning/HIV service on all the components of essential sexual reproductive health services with routine ART drug refilling and therapy in public health facilities before they are appointed for HIV services alone [18].

This study identified that 50% of current family planning users used integrated family planning/HIV services versus 6.1% who did not use integrated services. Further, 48.3% of those who had their need for family planning met were using integrated family planning/HIV services versus 32.7% who were not using integrated services. These findings were supported by a previous study conducted in Kenya which showed that 73% of women were more likely to use family planning if it was offered at the HIV clinic, and 45% reported to be using a barrier or natural method currently [19]. Therefore, the integrated family planning/HIV services in the ART clinics of Oromia Region, Ethiopia, should include counseling on available family planning services such as injectable contraceptive methods, pills, and condoms in the ART clinics. Moreover, women of reproductive age should be referred to the family planning room within the same facility for consultation on available long-acting and permanent contraceptive methods.

### Factors affecting the integration of family planning services with HIV services

This study identified the level of education, occupational status, residence, discussions with healthcare providers regarding family planning, fertility desire, having received counseling on family planning, and recent CD4 cells/ml^3^ as factors that increase the necessity for integrated family planning/HIV services. These findings are in line with previous studies conducted in Rakai by Brahmbhatt et al. [20] and a study conducted in Zambia by Hancock et al. [17]. Thus, it is critical to consider these identified factors for the implementation of integrated family planning/HIV services for women of reproductive age living with HIV attending ART/PMTC follow-up programs.

A previous study by Newmann et al. [19] identified factors that contributed to minimal integrated services as changes in contraceptive use and perception of others as being infertile, while this study did not support this finding. This may be because in this study almost all (97.1%) respondents preferred integrated sexual reproductive health and HIV services at the same facility or site. Additionally, almost all respondents, that is, 96.9%, preferred to receive sexual reproductive health with HIV services from the same providers. Another possible explanation may since more than 95% of respondents were satisfied with the utilization of integrated family planning/HIV services, which was confirmed during an exit interview at the time of the study.

### Limitation of the study

Though the problem of recall bias was minimized by conducting exit interviews; the current study is not free of social desirability bias in which some mothers may report the service as positive experiences while they are in the health facilities. As a strength, the study tried to cover a large number of health facilities including health centers and large sample size.

## Conclusion

This study established that overall; the integration of family planning/HIV services was relatively moderate among women of reproductive age living with HIV. The identified factors affected integration of family planning with HIV services were educational and occupational status, residence, discussion of family planning with healthcare providers, fertility desire, and CD4 counts (Additional file 1).

### Recommendation

- Engage women in the planning, implementation, and evaluation of the integrated family planning/HIV services to empower them to decide on their choices regarding family planning/HIV services.
- Promote the integrated family planning/HIV services using the mass media with local context in different languages.
- Develop and distribute tailored IEC/BCC materials (posters, leaflets, flyers, brochures, magazines) related to integrated family planning and HIV service to the community by using local languages for women of reproductive age and people living with HIV.
- FMOH and other stakeholders should renovate and equip health facilities with trained, motivated, respectful, caring, and compassionate healthcare providers to offer integrated reproductive health services—including family planning/HIV services—at single visits based on their needs.
- Healthcare providers (nurses, health officers, midwives, and physicians) should strengthen the provision of comprehensive health education throughout the sexual reproductive health services—including family planning and chronic care for HIV—in the waiting room area to increase awareness on the integrated people-centered family planning/HIV services in Oromia Region health care facilities.
- Provide quality counseling to improve the knowledge of reproductive-aged and empowered women by service providers on the integrated family planning/HIV services.
- The integration of family planning services with ART and PMTCT has a great contribution in achieving the end of new pediatric HIV infection. It is recognized in relevant policy statements, yet few PMTCT programs have increased access to contraception for HIV-infected women and couples who do not wish to become pregnant.
- A shift in how ART/PMTCT programs are conceptualized, implemented, and evaluated is needed to better address the family needs of HIV-infected women and accelerate progress towards ending new pediatric HIV infections.

## Supplementary Information

**Additional file 1.** German abstract.

## Data Availability

Datasets used in the current study are available from the corresponding author upon reasonable request.

## Abbreviations

AIDS: Acquired Immune Deficiency Syndrome
ART: Antiretroviral therapy
AOR: Adjusted odd ratio
CSA: Central Statistical Agency
CI: Confidence interval
COR: Crude odd ratio
EDHS: Ethiopian Demographic Health Survey
FHI: Family Health International
FMOH: Federal Ministry of Health
HIV: Human immuno-deficiency virus
IUDs: Intra uterine devices
OR: Odds ratio
ORHB: Oromia Regional Health Bureau
PMTCT: Prevention of mother-to-child transmission
SDGs: Sustainable development goals
STIs: Sexually transmitted infections
UNAIDS: United Nations Programme on HIV/AIDS
UNICEF: United Nations International Children's Emergency Fund
UNISA: University of South Africa
USAID: United States Agency for International Development
VCT: Voluntary HIV counseling and testing
WHO: World Health Organization
