# Supplementary material for: Integration of family planning services with HIV treatment for women of reproductive age attending ART clinic in Oromia regional state, Ethiopia
Source: Reprod Health. 2021 May 22;18:102. doi: 10.1186/s12978-021-01157-0 (PMC8141201; doi:10.1186/s12978-021-01157-0)
Supplement: Supplementary file 1 — Additional file 1. German abstract. [file 12978_2021_1157_MOESM1_ESM.docx]

Integration von Familienplanungsdiensten in die HIV-Behandlung von Frauen im gebärfähigen Alter, die die ART-Klinik im Regionalstaat Oromia, Äthiopien, besuchen

Dereje Bayissa Demissie 1 *, Prof. Rose Mmusi-Phetoe 2

1Department of Health Studies, Hochschule für Humanwissenschaften, Universität von Südafrika, Regionales Lernbüro Äthiopien und Millennium Medical College des St. Paul's Hospital, Addis Abeba, Äthiopien, * Korrespondenzautor, dereje.bayissa@sphmmc.edu.et

2Department of Public Health, Hochschule für Humanwissenschaften, Universität von Südafrika,

emphetrm@unisa.ac.za

ABSTRAKT

Hintergrund: In Umgebungen mit hoher HIV-Prävalenz ist das Management der sexuellen und reproduktiven Gesundheit entscheidend, um die HIV-Übertragung und die Müttersterblichkeit zu verringern. Die Integration der Familienplanung in HIV-Dienste ist für die HIV-Therapie, HIV-Prävention und -Pflege in einem Land mit begrenzten Ressourcen wie Äthiopien geeignet. Die Studie zielte darauf ab, den Status der Integration von Familienplanungsdiensten in die HIV-Behandlung und die Faktoren zu untersuchen, die mit der erfolgreichen Integration von Familienplanungs- und HIV-Diensten für Frauen im gebärfähigen Alter in Oromia, Äthiopien, verbunden sind, um bessere Gesundheitsergebnisse zu erzielen.

Methoden: Das Forschungsdesign dieser Studie war eine quantitative Umfrage, nicht experimentell, explorativ und deskriptiv. Ein Fragebogen wurde verwendet, um Daten von Frauen mit HIV zu sammeln, die ART-Kliniken in der Sonderzone der Umgebung von Finfinne, Region Oromia, in fünf Gesundheitszentren besuchen. Zur Auswahl von 654 Befragten wurden einfache Zufallsstichproben verwendet. Die Daten wurden mithilfe des Statistical Package for Social Sciences Version 23.0 analysiert. . Bivariate und multivariate logistische Regressionen wurden durchgeführt, um Faktoren zu identifizieren, die die Integration der Familienplanung mit HIV-Diensten mit der signifikanten Assoziation bei einem angepassten Odds Ratio (AOR) mit einem 95% -Konfidenzintervall (CI) zu kontrollierten Effekten möglicher Störfaktoren aus dem endgültigen Modell verbinden.

Ergebnis Die Rücklaufquote dieser Studie betrug 97,6% (654/670). Das Alter derjenigen, die die ausgefüllten Fragebögen beantworteten, lag zwischen 18 und 49 Jahren. Das Durchschnittsalter der Befragten betrug 31,86 Jahre mit einer SD von ± 6,0 Jahren. Die meisten Befragten in der Stichprobe waren in der Altersgruppe 26-35 (n = 374, 57%) und nur 96 (14,7%) in der Altersgruppe 18-25. Diese Gesamtintegration der FP-HIV-Dienste bei Frauen im gebärfähigen Alter, die mit HIV im regionalen Bundesstaat Oromia der Gesundheitszentren der Sonderzone leben, betrug 55,8%. .

Fast alle Befragten (n = 635, 97,1%) bevorzugten integrierte Familienplanung und HIV-Dienste von derselben Einrichtung und denselben Anbietern. Die Studie ergab, dass 622 (95%) mit der Inanspruchnahme integrierter Familienplanungs- / HIV-Dienste am zufriedensten waren.

Schlussfolgerung: Diese Studie ergab, dass die Integration von Familienplanung / HIV-Diensten bei Frauen im gebärfähigen Alter, die mit HIV leben, insgesamt relativ moderat war. Die identifizierten Faktoren, die die Integration der Familienplanung in HIV-Dienste beeinflussten, waren das Bildungsniveau, der berufliche Status und der Wohnort, die Diskussion der Familienplanung mit Gesundheitsdienstleistern, der Fruchtbarkeitswunsch und die CD4-Werte.

Schlüsselwörter: Familienplanung; HIV-Dienste; Integration; Frauen, die mit HIV leben
